# Supplementary material for: Implications of pre-diagnosis costs incurred by patients and their families for tuberculosis-related health-seeking behaviors in Mbeya and Songwe regions, Tanzania
Source: PLoS One. 2025 Nov 21;20(11):e0336270. doi: 10.1371/journal.pone.0336270 (PMC12637898; doi:10.1371/journal.pone.0336270)
Supplement: S1 Checklist — (DOCX) [file pone.0336270.s001.docx]

Inclusivity in global research

PLOS’ policy on inclusivity in global research aims to improve transparency in the reporting of research performed outside of researchers’ own country or community and ensures that PLOS publications reporting global research adhere to high standards for research ethics and authorship. Authors of relevant research articles may be asked to complete the questionnaire below, which outlines ethical, cultural, and scientific considerations specific to inclusivity in global research. This questionnaire may be requested when researchers have travelled to a different country to conduct research, if research uses samples collected in another country, research with Indigenous populations or their lands, or if research is on cultural artefacts. Researchers travelling to another country solely to use laboratory equipment will not normally be required to complete the questionnaire. However, the questionnaire can be requested at the journal’s discretion for any submission – if you have been requested to complete this questionnaire by the PLOS journal you submitted to, please do so.

Please complete the questionnaire below and include this as a Supporting Information file with your manuscript. Note that if your paper is accepted for publication, this checklist will be published with your article in the supporting information files. Please ensure that you reference the checklist in the main body of your manuscript. We suggest adding a subsection ‘Inclusivity in global research’ to your Methods section and adding the following sentence: “Additional information regarding the ethical, cultural, and scientific considerations specific to inclusivity in global research is included in the Supporting Information (SX Checklist)”

The questions have been designed to be applicable to a wide range of study types, and there are subsections for both human subjects research and non-human subjects research. If any of the questions are not relevant to your research please mark them as “N/A” as appropriate.

**Ethical considerations, permits and authorship**

*This section is applicable to all research types.*

Provide details as to who granted permissions and/or consent for the study to take place in the Methods section of your manuscript. This should include the names of **all** ethics boards, governmental organizations, community leaders or other bodies that provided approval for the study. If individuals provided approval refer to these people by their role or title but do not list their name(s).

In the Methods section of the manuscript page number 14, we describe the ethical approvals and permissions obtained for both the TB Sequel study and its qualitative sub-study. Specifically, ethical approval was granted by the Medical Research Coordinating Committee (MRCC) based on a protocol submitted to its sub-committee, the National Health Research Ethics Committee (NatHREC), operating under the National Institute for Medical Research (NIMR) in Tanzania. Additional permission was obtained from the Mbeya Medical Research and Ethics Review Committee, where the study was conducted.

The study was implemented in collaboration with local health authorities, and community engagement activities were conducted prior to the initiation of research to ensure transparency and secure community support. Informed consent was obtained from all individuals invited to participate, following the procedures outlined in the study protocol approved by the aforementioned national ethics authority.

If there were any deviations from the study protocol after approval was obtained please provide details of these changes in the Methods section of your manuscript.

There were no significant deviations from the approved study protocol during the implementation of the study. All procedures were conducted in full alignment with the protocol and supporting documents that were submitted to, and subsequently approved by, the MRCC in Tanzania, the designated authority for ethical clearance.

Did this study involve local collaborators that are residents of the country where the research was conducted or members of the community studied? If you do not have any authors from said communities, please provide an explanation for this below.

Yes, the study was conducted in two regions within Tanzania and involved participants who are residents and citizens of the country.

Everyone listed as an author should meet PLOS’ criteria for authorship and all individuals who meet these criteria should be included in the author byline, rather than the acknowledgements. For further information please see the journal’s Authorship Policy.

**Human subjects research (e.g. health research, medical research, cross-cultural psychology)**

Did you obtain written informed consent from a representative of the local community or region before the research took place? How did you establish who speaks for the community? Details of written informed consent obtained from study participants should be reported separately in the Methods section of your manuscript.

Permission was obtained from a recognized local ethics review body, the Mbeya Medical Research and Ethics Review Committee, which has jurisdiction over the southwestern highland regions of Tanzania, namely Mbeya, Songwe, Rukwa, Ruvuma, and Njombe. This ethical approval was clearly communicated to relevant subordinate offices, departments, and individuals during the process of seeking consent for study implementation in the respective regions

How did members of the local community provide input on the aims of the research investigation, its methodology, and its anticipated outcome(s)?

Members of the local community were engaged through consultations with community advisory boards (CABs) established at each study site. These boards included respected local leaders, former TB patients (TB Survivors), and representatives from community-based organizations. Their input was sought during the planning phase of the TB Sequel study and this was to ensure that the research’s aims, methodology, and expected outcomes aligned with community priorities and cultural considerations. Feedback from the CABs helped refine study materials, consent processes, and communication strategies to enhance understanding and trust within the community.

When engaging with the local community, how did you ensure that the informed consent documents and other materials could be understood by local stakeholders?

- The research findings will be shared with stakeholders in the community where the study was conducted through several channels to include;
- Community Meeting: the local meeting will be organized to present the key findings in a clear and accessible manner.
- A Summary Report: This will be summarizing the results and their implications, written in simple and easy-to-understand language, will be distributed to community representatives and other interested stakeholders.
- Publications: Academic publications resulting from the study will be made available to researchers and academician. In addition, a policy brief tailored for non-specialist audiences will be prepared to support broader understanding and potential policy use.

Will the findings of the research be made available in an understandable format to stakeholders in the community where the study was conducted (e.g. via a presentation, summary report, copies of publications, etc.)? Please provide details of how this will be achieved.

Feedback will be provided to all key stakeholders, including the communities where the study was conducted and the relevant authorities responsible for policy-making and the planning of health service interventions related to TB prevention, treatment, and control. To ensure the findings and recommendations are easily understood, the results will be communicated using clear, simple, and accessible language. Summarized reports will be shared with community representatives at the local level through appropriate channels, enabling broader dissemination to community members. Copies of the study’s publications will be distributed to regional and district-level authorities, including administrative bodies and committees or secretariats focused on health and TB-related issues.

The research team will also participate in locally organized workshops or conferences for research dissemination, where findings and recommendations will be presented through oral or poster formats. In addition, policy briefs will be developed and shared with departments and authorities involved in policy-making and implementation

**Non-human subjects research using specimens/ animals collected as part of the study, or those housed in archival collections. Examples include archaeology, paleontology, botany and zoology.**

As previously noted, permission to conduct the TB Sequel study was granted by the National Institute for Medical Research (NIMR) and Mbeya Medical Research and Ethics Review Committee. The approved study protocol included clear explanations of the potential benefits to participants, contingent on their informed consent. Among these benefits was the opportunity for the study’s findings to inform policy and management decisions related to TB prevention, treatment, and control—offering a pathway through which participants and their communities could ultimately benefit from evidence-based improvements in public health services. Before the commencement of data collection, informed consent was obtained from each individual approached for participation. Additionally, comprehensive explanations were provided to local authorities and community representatives regarding the study’s objectives, expected outcomes, and potential implications. All study procedures and agreements strictly adhered to established ethical guidelines and were aligned with relevant international standards for research involving human participants.

Did the permission you obtained from a local authority to perform the study include an agreement on access to outputs and benefit sharing? This may include procedures to enable fair distribution of the benefits and resources arising from the research performed. Please include any details of Prior Informed Consent and Benefit Sharing Agreements obtained. These may be required by field-specific regulations, for example the Convention on Biological Diversity (CBD) and the associated Nagoya Protocol.

If the material used in your study was imported, please A) provide the year it was imported and B) indicate whether permits were obtained to import/export the materials used, C) provide details of any permits obtained. If this information is not available, please indicate this.

The materials used in our study were not imported; all study materials and biological samples were obtained and processed locally within Tanzania

If you used archival specimens, please state how the material used in your study was acquired by the institute it is held in and provide details of any permits obtained for the original excavations/ sample collection. If this information is not available, please indicate this.

For the TB Sequel study, no archival specimens were used. All biological samples utilized in the study were collected prospectively as part of the approved study protocol.

How was the potential cultural significance of the materials collected in your study to local communities considered in your research design? Were Indigenous peoples and/or local researchers and institutions involved with archaeological excavations / collection of specimens? If so, please provide a description of their involvement.

The study did not involve the collection of materials with archaeological or cultural significance. The study focused on the collection of clinical and biological samples related to tuberculosis diagnosis and treatment.

If your manuscript includes photographs of human remains please indicate whether authors obtained permission from descendants or affiliated cultural communities to do so.

The manuscript resulting from the TB Sequel study does not include any photographs of human remains**.**
