# Supplementary material for: Implications of pre-diagnosis costs incurred by patients and their families for tuberculosis-related health-seeking behaviors in Mbeya and Songwe regions, Tanzania
Source: PLoS One. 2025 Nov 21;20(11):e0336270. doi: 10.1371/journal.pone.0336270 (PMC12637898; doi:10.1371/journal.pone.0336270)
Supplement: Attachment — Submitted filename: Response to Reviewers.docx [file pone.0336270.s002.docx]

National Institute for Medical Research

P.O. Box 9653, Dar es Salaam, Tanzania

Office Phone: +255-22-2121400

Telephone number: +255-782-007414

Email address: stellakilima@yahoo.com

09^TH^ July, 2025

Bilal Ahmad Rahimi, M.D., D.T.M.&H., M.C.T.P., Ph.D
Academic Editor
PLOS ONE

Dear Editor

REF: RE-SUBMISSION OF THE MANUSCRIPT TITLED “**Implications of pre-diagnosis costs incurred by patients and their families for tuberculosis-related health-seeking behaviors in Mbeya and Songwe regions, Tanzania”**

The heading above is of concern,

I am writing to inform you that I am resubmitting the manuscript titled *"Implications of pre-diagnosis costs incurred by patients and their families for tuberculosis-related health-seeking behaviors in Mbeya and Songwe regions, Tanzania"* for consideration in PLOS ONE.

We have carefully addressed all the reviewer comments and have made the necessary revisions to improve the quality of our manuscript. The revisions focus on enhancing the clarity of our findings, providing additional analyses, and incorporating the valuable feedback received. A detailed list of the changes made, along with our responses to each reviewers’ comments, is included in the attached document. We would also like to clarify that the funder had no role in the study design, data collection and analysis, decision to publish, or preparation of the manuscript.

We sincerely hope that the revised manuscript meets the journal’s standards for publication, and we appreciate the time and effort that the reviewers and editors have put into evaluating our work.

Thank you very much for your continued consideration of our submission. Please do not hesitate to contact me if further information or clarification is required.

Sincerely,

Stella Peter Kilima

**National Institute for Medical Research**

|  | **Comment** | **response** | **Page number** | **Lines number** |
| --- | --- | --- | --- | --- |
|  | We note that Figures 1 and 2 in your submission contain [map/satellite] images which may be copyrighted. All PLOS content is published under the Creative Commons Attribution License (CC BY 4.0), which means that the manuscript, images, and Supporting Information files will be freely available online, and any third party is permitted to access, download, copy, distribute, and use these materials in any way, even commercially, with proper attribution. For these reasons, we cannot publish previously copyrighted maps or satellite images created using proprietary data, such as Google software (Google Maps, Street View, and Earth).  We require you to either (1) present written permission from the copyright holder to publish these figures specifically under the CC BY 4.0 license, or (2) remove the figures from your submission. | Thank you for your guidance regarding the copyright requirements for Figures 1 and 2 in our submission.  In compliance with PLOS ONE's licensing policy and the CC BY 4.0 requirements, we have removed Figures 1 and 2 from the manuscript. We understand the importance of ensuring that all published content is fully compatible with the journal’s open-access licensing terms.  We have updated the manuscript accordingly |  |  |
|  | **Reviewer 1** |  |  |  |
| 1 | The manuscript would benefit from additional editing to harmonize terminology and be more concise and to the point. | Thank you for your valuable feedback. We appreciated your suggestion for additional editing to harmonize terminology and improve conciseness. We carefully reviewed the manuscript to ensure consistent terminology throughout and revised the text to make it more concise and to the point, while maintaining clarity and accuracy in the content. Your input helped strengthen the overall quality of the manuscript. | Whole document |  |
| 2 | State "mixed methods study" clearly in Methodology  References to global and national TB burden source from various Global TB reports between 2019-2023. Please avoid this pick-and-choose and stick to the latest data. | Thank you for your constructive feedback. In response, we clearly stated that the study employed a mixed methods approach in the Methodology section to enhance clarity.  We have revised the Methodology section to clearly state the mixed methods study. The revised sentence now reads: *“This study employed a mixed methods approach, incorporating both quantitative and qualitative components*  Additionally, we ensured that all references to the global and national TB burden were based on the latest available data, drawn from Global TB reports between 2019 and 2023, to avoid inconsistencies. We appreciated your suggestion and made the necessary adjustments to improve the manuscript | Page 2 and 7 | Lines 30; 141-142 |
| 3 | Several aspects missing: - The COVID-19 impact on the situation/study | Thank you for your valuable feedback. In response, we included a discussion on the impact of COVID-19 on the study, acknowledging its potential effects on the results and the broader context. We addressed how the pandemic may have influenced the TB burden and any changes in data patterns, ensuring that this aspect is thoroughly covered in the revised manuscript | Result section Page 24  Discussion section page 28  Study limitations page 31 | Lines 472-481  Lines 569-576  Lines 635-644 |
| 4 | - Definition of catastrophic cost and current estimated prevalence (WHO: 49%) | Thank you for providing a more detailed explanation. We revised the manuscript to incorporate your precise definition of catastrophic costs. Here's a refined version of the definition:  "Catastrophic costs occur when total costs of TB care seeking including direct medical, direct non medical and indirect costs exceed 20% of household’s annual income prior to TB diagnosis  This definition and prevalence are clearly articulated in the manuscript, with appropriate citation of the WHO reference. Thank you again for highlighting this point. | Page 5, Background section | Lines 92-94 |
| 5 | central to this manuscript - Context of costs incurred - value against benchmark (average weekly, monthly or annual income.) Some statements are incorrect, open to misinterpretation, or miss-quoted: - Tanzania,[...] has high cases of TB in comparison to other countries within Africa and globally - better stick to is one of the 30 high TB burden countries, as there are other countries with much higher TB burden in Africa & globally | We appreciate the reviewer’s emphasis on contextualizing the costs incurred by TB patients. In response, we have updated the **literature review section** to include comparative data from other African countries, thereby highlighting the substantial share of pre-diagnosis costs in relation to household income and time loss. Additionally, in the **discussion section**, we now provide a clearer comparison of pre-treatment costs against the national minimum monthly wage to better illustrate the financial burden, particularly for low-income populations  Regarding the statement on TB burden, we appreciate your clarification. We have revised the wording to align with WHO classifications and now state:  “Tanzania is one of the 30 high TB burden countries globally” | Page 4 &5; Literature review section  Discussion section  Background section 4 | Lines 75-83  Lines 558-568  Lines 100-101 |
| 6 | - A recent national TB prevalence survey in Tanzania revealed that 145 out of every 100,000 tuberculosis cases were detected - This is a miss-quote – the case detection rate is 145/100,000 population not 145 out of 100,000 tuberculosis patients | Thank you for pointing out the misquote. We apologize for the error in phrasing. We have revised the statement to correctly reflect that the case detection rate for tuberculosis in Tanzania is 145 per 100,000 population, not per 100,000 tuberculosis patients. We appreciate your careful attention. | Page 6 Background section | Lines 104-106 |
| 7 | Four patients initiated a MDR regime because they were Multidrug-resistant (MDR). - People are not MDR-TB, they have MDR-TB. Please ensure non-stigmatising respectful language | Thank you for the important feedback. We fully agree with the need for respectful and non-stigmatizing language. The sentence has been revised to:  "Four patients initiated a MDR-TB regimen because they had multidrug-resistant tuberculosis (MDR-TB)."  We appreciate your guidance on this and have made the necessary adjustments to ensure respectful language throughout the manuscript. | Page 15 Result section | Lines 313-315 |
| **8** | **Methodology:** The criteria for the sampling strategy are missing, as are sampling bias mitigation. | Thank you for your valuable comment. We apologize for the oversight. We have now added a detailed explanation of the criteria used for our sampling strategy in the revised manuscript. Specifically, we clarify the following aspects of our sampling approach:  The study employed random sampling, to ensure representative selection. Additionally, we have addressed potential sampling biases and have outlined the strategies employed to minimize them.  For qualitative sampling, purposively selection were used. | Page 8 Methodology section | Lines 160-165 and 184-186 |
| 9 | Missing insight into how many people have declined participation. | Thank you for your comment. A total 319 participants were initially recruited. Of these, 49 did not meet the eligibility criteria, 6 tested negative for TB, and 3 declined to proceed with follow-up procedures. Consequently, 261 participants with laboratory-confirmed PTB patients, representing 81% of those initially recruited, were enrolled in the TB Sequel study | Page 15 result section | Lines 301-305 |
| 10 | Why were the questionnaires orally translated rather than fully translated in writing (ensuring consistency of translation)? | Thank you for your observation. We acknowledge the distinction in translation approaches between the **quantitative and qualitative** tools. The quantitative questionnaire was administered through oral translation by trained bilingual researchers to accommodate real-time interview conditions across diverse settings. In contrast, the qualitative interview guide was fully translated into written Kiswahili to ensure consistency in probing and depth of discussion across all interviews. This written translation was independently verified by two bilingual researchers for accuracy and reliability. | Quantitative Page 10 methodology section  Qualitative Page 11 methodology section | Lines 197-200  Lines 221-223 |
| 11 | Table 5. Themes & quotes from participants: It would be better to add the quotes to the respective thematic paragraphs to underLines the stated “findings”/ interpretations At present the format is a bit disjointed and previously stated “findings” are missing the actual data/ information/ quotes underpinning them Discussion: reference some more countries | Thank you for this helpful suggestion. We agree that integrating the quotes directly into the thematic paragraphs enhances the clarity and supports the findings more effectively. In response to your feedback, we have revised the manuscript by embedding the relevant quotes within the corresponding thematic sections, ensuring that the quotes directly align with the findings and interpretations. This change aims to create a more cohesive structure and provide a clearer link between the qualitative data and the themes discussed. We hope this improves the readability and coherence of the analysis. | Results section (qualitative) | Lines 376-526 |
|  | **Reviewer 2** |  |  |  |
| 11 | The reference about OOPs are all related to diagnosed TB cases rather than pre-diagnosed cases. Is there no data on costs associated with pre-diagnosis PTB? | Thank you for your comment and observation. You’re correct that many references on OOP costs tend to focus on diagnosed TB cases. However, in several studies, **pre-treatment and on-treatment costs are often combined** and reported together as part of the overall financial burden, particularly when calculating **catastrophic costs**. This makes it challenging to find studies that isolate costs strictly associated with the pre-diagnosis phase. | Background section | The whole section |
| 12 | Please find some references for this statement: “While many high TB burden countries offer essential TB diagnosis and treatment for free... | Thank you for your valuable feedback. I appreciate your suggestion to provide references for the statement: However, the statement has been revised to read “*Although TB diagnosis and treatment are provided free of charge in many public health systems, patients often incur substantial out-of-pocket expenses, especially during the pre-diagnosis phase of care*” The following references was used to inform this statement.  Assefa DG, Dememew ZG, Zeleke ED, Manyazewal T, Bedru A. Financial burden of tuberculosis diagnosis and treatment for patients in Ethiopia: a systematic review and meta-analysis. BMC Public Health. 2024;24(1):1–14.  WHO. Global tuberculosis report 2023. Geneva: World Health Organization; 2023. Licence: CC BY-NC-SA 3.0 IGO; 2023 | Page 4, Background section | Lines 66-68 |
| 13 | The motivation for the paper could be strengthened. In the second paragraph of the Background, the authors state that the economic burden of PTB is significant, but provide no reference or quantification of their claim. They provide a reference based on Thailand only for OOPs, while there are various better sources to back up this statement of the economic burden of PTB, that could include Africa as well. I would urge the authors to strengthen this point, as it is crucial to motivating their paper. | Thank you for your valuable feedback. We agree that providing stronger support for the claim regarding the economic burden of pre-diagnosis PTB is crucial for motivating the study. In response to your comment, we have expanded the background section to include more references and data quantifying the economic burden of PTB, particularly with a broader focus on regions such as Africa.  The following references were used to inform: Litvinjenko S, Magwood O, Wu S, Wei X. Burden of tuberculosis among vulnerable populations worldwide: an overview of systematic reviews. Lancet Infect Dis. 2023;23(12):1395–407.  Assefa DG, Dememew ZG, Zeleke ED, Manyazewal T, Bedru A. Financial burden of tuberculosis diagnosis and treatment for patients in Ethiopia: a systematic review and meta-analysis. BMC Public Health. 2024;24(1):1–14.  WHO. Global tuberculosis report 2023. Geneva: World Health Organization; 2023. Licence: CC BY-NC-SA 3.0 IGO; 2023.  Asres A, Jerene D, Deressa W. Pre- And post-diagnosis costs of tuberculosis to patients on Directly Observed Treatment Short course in districts of southwestern Ethiopia: A longitudinal study. J Health Popul Nutr. 2018;37(1):1–11.  Isangula K, Philbert D, Ngari F, Ajeme T, Kimaro G, Yimer G, et al. Implementation of evidence-based multiple focus integrated intensified TB screening to end TB (EXIT-TB) package in East Africa: a qualitative study. BMC Infect Dis. 2023;23(1):1–17.  Jarde A, Romano E, Afaq S, Elsony A, Lin Y, Huque R, et al. Prevalence and risks of tuberculosis income and multimorbidity in low- ­ income countries : a meta- ­ review. BMJ Open. 2022;1(12(9):e060906).  Devoid I, Sillah AK, Sutherland J, Owolabi O, Ivanova O, Govathson C, et al. The household economic burden of drug-susceptible TB diagnosis and treatment in The Gambia. International Journal of Tuberculosis and Lung Disease. 2022 Dec 1;26(12):1162–9. | Page 4 Background section | Lines 65-83 |
| 14 | I think that the background needs to be recast to reflect the focus of the paper on economic costs associated with pre-diagnosis PTB. In its current form, it is a mix of costs experienced before, during and post diagnosis. Is there an argument for pre-diagnosis costs? If so, a specific motivation for pre-diagnosis economic costs should be presented in the background. | Thank you for your thoughtful comment. We acknowledge that the current background section was not sufficiently focused on the specific topic of pre-diagnosis economic costs associated with pulmonary tuberculosis (PTB), and that it blends costs incurred before, during, and after diagnosis. In light of your feedback, we have revised the background to more clearly emphasize the rationale for focusing on pre-diagnosis costs. | Page 4-7 Background section | Lines 55-138 |
| 15 | The study needs to do better job at describing why PTB pre-treatment costs are important. What do we know about them, why do we need to care? | Thank you for your insightful comment. We agree that the importance of pre-treatment costs for PTB needs to be more clearly articulated. In response, we have revised the manuscript to better emphasize the significance of these costs and the reasons they warrant attention. | Page 7, Background section | Lines 128-138 |
| 16 | The authors say that “prevalence survey in Tanzania revealed that 145 out of every 100,000 tuberculosis cases were detected”. Is this per TB cases or per population? | Thank you for your valuable comment. We appreciate the opportunity to clarify this point.  We have revised the statement to correctly reflect that the case detection rate for tuberculosis in Tanzania is 145 per 100,000 population, not per 100,000 tuberculosis patients. This figure represents the number of people diagnosed with TB per 100,000 members of the general population, as reported in the prevalence survey. | Page 6 Background section | Lines 104-106 |
|  | And how is progress being assessed with one data point? I am sure that authors know something about the **trajectory** of TB burden in Tanzania. Let them clarify this point. | Regarding the assessment of progress with a single data point, we recognize that it would be insufficient to gauge the trajectory of TB burden based solely on one prevalence survey. We clarified that the cited data represents the most recent available point in time for the survey, but we also acknowledge that TB burden should be tracked over time to understand progress or setbacks. In the revised manuscript, we have added a brief discussion of the trends in TB burden in Tanzania, based on available data from National TB and Leprosy Programme | Page 6 Background section | Lines 100-108 |
|  | I would also urge the authors to provide further details about where TB services are delivered in Tanzania. They have said that it is in the public sector. But, no details about the level of care, referral system, level of access (what percentage of patients receive care in the public system, etc.), quality, etc. | Thank you for your helpful suggestion. We appreciate your recommendation to provide more details regarding to where TB services delivered in Tanzania. To address this, we have expanded the manuscript to include more information about the public healthcare system's role in TB care. | Page 6; Background section | Lines 109-118 |
| 18 | Do all the health facilities have capacity to diagnose PTB? In the analysis, it is shown that patients incurred significant expenses while visiting various providers, partly because they were unaware which provider had the capacity to diagnose them. | Thank you for the comment. We have clarified that not all health facilities in Tanzania are equipped to diagnose PTB. A national survey shows that limited diagnostic capacity and lack of patient awareness contribute to delays in diagnosis and increased out-of-pocket expenses due to multiple consultations. | Page 6; Background section | Lines 109-118 |
| **19** | **Methods** The patients were followed up over a relatively long period of time. A key concern I have is that given that data collected retrospectively over time, what is the potential effect of recall bias? | Thank you for your insightful comment regarding the potential for recall bias. We would like to clarify that this was not a retrospective study. Data collection was conducted prospectively at the enrolment visit before any study interventions, using an adapted version of the WHO patient cost survey. Patients were asked to recall their care-seeking journey from symptom onset to diagnosis, which had a relatively short median recall period of approximately 8 weeks (IQR 4–16 weeks). We also employed structured recall techniques such as time anchoring to support accurate recall. Given this design and the limited recall window, we believe the impact of recall bias on the data is minimal. | Page 11; Methodology section | Lines 212-219 |
| 20 | What was the median period in months or weeks, between onset of symptoms to initiation of treatment? How long was the diagnosis? | Thank you for your comment. The data you mentioned has already been included in the revised manuscript, specifically in **Table 3.** The median time from symptom onset to treatment initiation is IQR 8 (4.0-16.0) is presented, along with a categorization of these data. | Page 18 results section | Lines 340-343 |
| 21 | What were the inclusion and exclusion criteria for the selection of subjects, apart from age and TB test? | Thank you for your comment and for highlighting the need for clarity regarding participant selection. In addition to age and TB test results, we have now added a detailed description of the inclusion and exclusion criteria for both the qualitative and quantitative components of the study in the Methods section. | Page 9 Methodology | Quantitative, Lines 170-183  Qualitative, Lines 184- 193 |
| 22 | From which health facilities were these patients being referred from? | Thank you for your comment. The TB patients included in the study were referred from a total of 24 randomly selected facilities that provide TB care and treatment. To ensure that the selected patients met the criteria of being TB positive, they were referred from the selected 13 hospitals, 10 health centers, and one dispensary. The facility were selected among the TB facility that accredited to provide both TB diagnosis and treatment | Page 8 methodology section | Lines 148- 158 |
| 23 | It would be interesting, if possible, for the authors to run a multivariate analysis of costs per patient characteristics. Does sex, HIV status, residential location, lengthy of delay in seeking treatment, etc., have anything to do with the level of reported costs? | Thank you for your thoughtful suggestion regarding the potential for a multivariate analysis of costs by patient characteristics such as sex, HIV status, residential location, and delay in seeking treatment. We agree that such analyses are often useful for identifying factors associated with different outcomes. However, in this study, the outcome of interest, pre-treatment costs reflects the period before baseline (i.e., from symptom onset to TB treatment initiation). Since many baseline characteristics (such as age, sex, and HIV status) were recorded at or after treatment initiation, we are cautious about using them as explanatory variables for an outcome that occurred prior to their measurement. In epidemiological terms, this raises concerns about temporality and potential reverse causality, which could compromise the validity of the analysis. |  |  |
| 24 | Could the authors comments on what are these drugs that patients taking pre-diagnosis? | Thank you for your question. We appreciate the opportunity to clarify the medications that patients may be taking prior to receiving an official TB diagnosis.  In the pre-diagnosis phase, many qualitative participants said patients with symptoms of pulmonary tuberculosis (PTB) prefer to start with self-medication. purchase over-the-counter remedies such as cough syrup, pain killers and antibiotic, like amoxicillin. | Page 21; result section | Lines 409-416 |
| 25 | Given that the data were gathered over a period of time, it would have been helpful to see the economic costs calculated over a specified period of time, say per month or six months, rather than aggregated over the entire sample. That would also provide a quality check of the potential recall bias that I talked about earlier | Thank you for your insightful comment. In our study, we utilized the WHO patient cost survey tool, which collects data on direct expenses and income lost from the onset of symptoms up to enrolment/start of TB treatment. The median duration for this pre-treatment period is approximately 8 weeks (IQR 4–16 weeks), as reported in **Table 3**. Since these costs are based on each patient’s unique care-seeking journey and number of providers visited, the expenditures are not evenly distributed over time. Therefore, dividing total pre-treatment costs into fixed monthly amounts would not accurately reflect the true distribution of expenses. Moreover, Reporting costs per fixed time intervals such as monthly or six-monthly is generally more appropriate for **on-treatment cost**s, where treatment phases tend to be more standardized in duration across patients. We believe that aggregating pre-treatment costs as we have done provides a more valid and meaningful representation of the economic burden during this variable and often unpredictable phase. |  |  |
| 26 | I would suggest to strengthen the analysis by putting these costs into some perspective for ease of interpretation. For example, how do they relate to per capita income, | We appreciate the clarification. We acknowledge the distinction between economic, financial, and patient/household costs. As per your suggestion, we have revised the manuscript to refer to "patient costs" or "household costs" rather than "**economic costs,"** to more accurately reflect the nature of the expenditures reported—namely, direct out-of-pocket expenses and time-related costs borne by the patient and household. But in the discussion section we managed to discuss the Relationship between TB related costs and household monthly income. | Page 28 Discussion section | lines 553-568 |
| **27** | **Discussion** This section needs some attention to provide a clear storyline about economic costs of PTB pre-diagnosis. In its current form, the discussion does not communicate very well the interpretation of the study findings. As an example, I am not cure what is meant by the following statements: “Therefore, regrets arise after individuals or their households/families opt to use medicines that are obtained outside of formal prescriptions by qualified medical officers, resulting in certain monetary and non-monetary costs.”, and also “Opting for shortcuts in TB care-seeking behaviours is a challenge that is faced in both studied regions. Initially, the respective parties believe that by opting for shortcuts in TB care seeking, they can continue with their daily activities, such as participating in economically productive work as farmers or other jobs that provide a means of subsistence”. What exactly are these shortcuts? | Thank you for your valuable comments. We agree that the discussion could be clearer in its interpretation and communication of the study’s findings, particularly regarding the points you have highlighted. We have revised the discussion section to improve the clarity and coherence of the narrative and to ensure a better understanding of the economic costs related to pre-diagnosis PTB.  To clarify the first statement about the use of non-prescribed medications, now read as;  *As a results individuals often resort to self-medication or consult informal healthcare providers, both of which can lead to delayed diagnosis and higher cumulative costs*.  We have carefully revised the entire section to provide a more coherent narrative that clearly links patient behavior, healthcare access barriers, and the resulting economic burden. We believe these revisions now present a clearer and more accurate interpretation of the study’s findings. | Page 27 discussion section | Lines 538-540  The whole discussion sectio2 |
| 28 | The authors could also provide further discussion of the policy implications of these findings. They should attempt to link policy implications to the specific findings of their study. In several parts, this discussion is sounding more speculative than rooted in the findings of the study. As an example, I did not see where this recommendation came from in the analysis: “Additionally, national TB control programs should make necessary adjustments, such as designing and institutionalising social protection options to support the less privileged and marginalised individuals and their families”. | Thank you for this valuable feedback. We appreciate your point about linking the policy implications more clearly to the specific findings of the study. We understand that the recommendations should be based on the data and not sound speculative. We have revised the discussion to ensure that all policy recommendations are directly tied to the study's findings and are more clearly rooted in the data we presented. | Page 29; Discussion section | Lines 590-602 |
| 29 | What are the implications for strengthening key aspects of the TB in Tanzania: notification, screening, detection, treatment, etc. This paper is about implications, perhaps more attention could be given to the health system side of things. | Thank you for your valuable suggestion. We agree that the health system's role is crucial to addressing the economic burden of TB, and we appreciate your insight into the need to focus more on strengthening key aspects of the TB control program in Tanzania.  In response, we have revised the discussion to more clearly highlight the implications for strengthening various components of the TB control system, particularly in the context of the economic challenges faced by patients during the pre-diagnosis phase. | Page 29; Discussion section | Lines 582-589 |
| 30 | **Conclusion** I think that this section needs to be re-written and shortened. The focus should be the evidence found in terms of the answer to the question posed or objective. What is the scale or scope of economic costs of PTB in Tanzania? Some of the points in the second and third paragraphs could go to the discussion section. | Thank you for this helpful feedback. We agree that the conclusion should be more focused and succinct, emphasizing the key findings related to the economic costs of PTB in Tanzania. We have revised the conclusion to streamline it and directly address the objective of the study: to understand the scale and scope of the economic burden of pre-diagnosis PTB. | Page 32 | 25 Lines 653-663 |
| 31 | **Minor** comments This sentence, However, its burden is higher in specific countries, Tanzania being a no-exclusion, could be simplified to read: However, its burden is higher in specific countries, Tanzania being one such country. Writing style needs some attention. For example, use more conventional language for this statement: “As mentioned above, the public health implications of TB are dire when examined both broadly and in depth.” Terms such as dire, immense, etc., are not conventional scholarly words in scientific writing. | Thank you for pointing this out. We recognize the importance of maintaining a formal and scholarly tone throughout the manuscript.  *The sentence revised to read as, “Pulmonary tuberculosis (PTB), remains one of the leading causes of death from infectious diseases worldwide, its burden is disproportionately affecting low- and middle-income countries (LMICs), including Tanzania.* | Abstract section Page 2 | Lines 24-26 |
| 32 | Would the authors want to clarify or simply this sentence: “Therefore, in the absence of some safety net mechanisms, such as user fee exemptions or health insurance, patients and their families find themselves in access to care financial hardships, contrary to the UHC goal and the global End-TB Strategy” | Thank you for your valuable feedback. We agree that the original sentence required clarification for improved readability and impact. This section has been revised to enhance clarity and precision. |  |  |
