# Supplementary material for: Implications of pre-diagnosis costs incurred by patients and their families for tuberculosis-related health-seeking behaviors in Mbeya and Songwe regions, Tanzania
Source: PLoS One. 2025 Nov 21;20(11):e0336270. doi: 10.1371/journal.pone.0336270 (PMC12637898; doi:10.1371/journal.pone.0336270)
Supplement: Attachment — Submitted filename: RESPON~2.DOC [file pone.0336270.s003.DOC]

National Institute for Medical Research

P.O. Box 9653, Dar es Salaam, Tanzania

Office Phone: +255-22-2121400

Telephone number: +255-782-007414

Email address: stellakilima@yahoo.com

03^rd^ October, 2025

Bilal Ahmad Rahimi, M.D., D.T.M.&H., M.C.T.P., Ph.D
Academic Editor
PLOS ONE

Dear Editor

REF: RE-SUBMISSION OF THE MANUSCRIPT TITLED “**Implications of pre-diagnosis costs incurred by patients and their families for tuberculosis-related health-seeking behavior in Mbeya and Songwe regions, Tanzania”**

The heading above is of concern,

I am writing to inform you that I am resubmitting the manuscript titled *"Implications of pre-diagnosis costs incurred by patients and their families for tuberculosis-related health-seeking behaviors in Mbeya and Songwe regions, Tanzania"* for consideration in PLOS ONE.

We have carefully addressed all the reviewer comments and have made the necessary revisions to improve the quality of our manuscript. The revisions focus on enhancing the clarity of our findings, providing additional analyses, and incorporating the valuable feedback received. A detailed list of the changes made, along with our responses to reviewers’ comments, is included in the attached document. We would also like to clarify that the funder had no role in the study design, data collection and analysis, decision to publish, or preparation of the manuscript.

Thank you very much for your continued consideration of our submission.

Sincerely,

Stella Peter Kilima

**National Institute for Medical Research**

**Reviewer 1**

1. Costs are relative to income; this is why the WHO is defining catastrophic costs as “exceeding 20% of annual household income or expenditure” – therefore the discussion (and the abstract) would benefit from an expression of costs as percentage of the annual income or expenditure. It is also not clear from the manuscript if the USD 28.8 denotes the average total expenditure or the average expenditure per month. Line 41 in the abstract speaks of “average total pre-treatment cost” while line 562 denotes “cost incurred by patients in this study was USD 28.80 per month” Furthermore, there seems to be a typo 28.3 instead of the 28.8 (Table 4 shows a mean of USD 28.8 – so the number in line 41 should be corrected.
Line 41) expression “USD 28.3 (representing x% of average/ minimum annual income)” would be providing helpful context for the reader

**Response:** Thank you for this insightful comment. We agree that expressing costs as a percentage of annual income or expenditure can provide valuable context. However, due to the high levels of unemployment observed in our study population (now reflected in Table 2), and the fact that the majority of participants experienced delays in seeking care ranging from 4–8 months with a few delaying up to 16 months, we opted to calculate costs based on the minimum monthly income. This decision was informed by the observation that most respondents reported earning either the national minimum wage or an amount close to it. We chose not to use average annual income as a reference point, as this could be misleading in our context and potentially underestimate the financial burden of pre-treatment costs. For individuals with limited or unstable income, an expenditure of USD 28.80 represents a disproportionately large burden compared to someone with regular and stable earnings.

**Page 2 Line 40: Page 21; Line: 377-382; Page 29&30 line 573-597; Page 34 Line 685-689;**

**2. Comment:** For completeness and transparency, it would be helpful to describe in one sentence what the Sequel study is about. Also to understand that no inadvertent sample bias is included by working with participants selected for another study e.g. asking participants to travel to another health facility for additional interventions, when they have already incurred increased pre-diagnostic costs and delays does this influence willingness to participate?

**Response:** Thank you for this valuable observation. We have now added a brief description of the Sequel study in the Methods section to improve clarity and context. We also acknowledge the potential for selection bias and have clarified in the revised manuscript how participants were recruited.

**Page 8; Line 143-145; Page 9: Line 166-178; Page 10 line 193-197; Page 34; line 672-682;**

3. Spelling & grammar: The document needs some more work on the copyediting: please carefully check and edit the manuscript as there are still large numbers of spelling and grammar mistakes which might not be picked up by the automated checker (as they are proper words) but distort the meaning of your sentences. Some (not exhaustive) examples are:

Line 28) grammar: suggest removing “were”
Line 45) L missing in “formal”
Line 95) spelling “dater” instead of “deter”
Line 143) please spell out “Sub-Saharan African” on first use
Line 303) please remove “patient” from the end of the line – seems a remnant from editing and is not needed
Line 331)/2) sentence is confusing – why not simply say “have not contacted any other service providers before presenting to the health facility”?
Line 396) Missing “who” after “Among those”

**Response:** Thank you for your detailed feedback regarding spelling and grammatical issues in the manuscript. We appreciate you pointing out specific examples, and we acknowledge that clarity is critical to ensure the manuscript’s meaning is not distorted. We have now undertaken a thorough manual copyediting of the manuscript beyond automated spell checks, addressing both the examples you noted and other issues throughout the text. The specific corrections you highlighted have been implemented as follows:

- Line 27: Removed the unnecessary word “were” to improve sentence structure.
- Line 44: Corrected the spelling of “formal” (previously missing “L”).
- Line 94: Corrected the misspelling of “deter” (previously written as “dater”).
- Line 146: “Sub-Saharan African” has now been fully spelled out on first mention for clarity.
- Line 313: Removed the redundant word “patient” at the end of the sentence.
- Line 338–339: Revised the sentence for clarity to read: “...had not contacted any other service providers before presenting to the health facility.”
- Line 403 Inserted “who” after “Among those” to complete the sentence grammatically.

We have also carefully reviewed the rest of the manuscript to ensure that similar errors have been corrected and the overall language has been improved for clarity and accuracy.

4. Stigmatising language: Line 164) please mitigate stigmatising language HIV-positive => People/ participants living with HIV; diabetic patients => people/ participants with diabetes mellitus

**Response:** Thank you for highlighting the use of potentially stigmatising language. We fully agree with the importance of using person-first, respectful terminology in all scientific writing. We have now revised the manuscript to replace phrases such as “HIV-positive” and “diabetic patients” with more appropriate alternatives, including “people living with HIV” and “people with diabetes mellitus.” We have reviewed the entire manuscript to ensure consistent and respectful language is used throughout.

Page 6 Line114-115; **Page 8 Line 166-167; Page 29 Line 560; Page 16 Line 335-336**

5. Inclusion criteria: Line 189) it strikes me that the ability to comfortable communicate in English or Kiswahili and provide written consent might exclude people with less or no formal education and thus potentially less socio-economic resources/ security - i.e. at higher risk of being affected by catastrophic costs, thus potentially underestimating the true OOP expenditures. Has this been considered and mitigated? It should at least be acknowledged.

**Response:** Thank you for this important observation. You are absolutely right to highlight the risk of excluding individuals with lower levels of formal education or literacy, which could potentially introduce bias and underestimate out-of-pocket (OOP) expenditures. We would like to clarify that one of the inclusion criteria was the ability to communicate in either Kiswahili or English, depending on the participant’s preference. In Tanzania, Kiswahili is the national language and widely spoken across the population. While most participants chose to communicate in Kiswahili, a few preferred English, and they were not excluded from the study. Furthermore, individuals with low literacy or who were unable to read or write were also not excluded. In such cases, the informed consent process was conducted verbally, with the entire consent form read aloud to the participant. Consent was then obtained using a thumbprint, in the presence of an impartial witness, in accordance with ethical approvals and national research guidelines.

**Page 10 Line 198-203**

**6:** Table 1: Line 285/286) the table would benefit from proper lines to better distinguish which sub-themes belong to which theme.

**Response:** Thank you for this helpful suggestion. We have revised Table 1 to include clearer visual formatting, including horizontal lines and indentation where appropriate, to better distinguish sub-themes from main themes. This improves the readability and clarity of the table, making it easier to follow the thematic structure of the findings.

**Page 15 line 295**

7. P-values Lines 320-322) “Other variables, such as age, sex, education level, HIV status, smear microscopy results, and treatment regimen, show no significant differences in pre-treatment costs, based on the p-values calculated in the tests (Table 2)” - the sentence refers to p-values in table 2 – which do not exist in said table please rephrase

**Response**: Thank you for this helpful observation. We acknowledge the oversight and we revised the table accordingly. The sentence has been rephrased to accurately reflect the contents of the updated table, which now includes the p-values for comparisons of pre-treatment costs across the listed variables. **Page 17; Line 330-333**

8. Figure 1: Line 337) Figure 1 would benefit from clearer labelling of the x axis (what do the numbers 1-4 denote?) and some more description. As the values are percentages of visits in each category, but the number of people in each group likely decreases is the figure not slightly misleading? I would at least expect a number of respondents indicated for each group

**Response:** Thank you for this helpful comment. We agree that Figure 1 would benefit from clearer labeling and additional contextual information. We have revised the x-axis labels to clarify what the numbers 1–4 represent, and we have updated the figure caption to explicitly describe the figure content. To address the concern regarding potentially misleading interpretation due to decreasing sample sizes across visit categories, we have now included the **number of respondents (n)** in each group within the figure itself and clarified this in the caption. This will allow readers to better interpret the percentages shown in relation to the underlying group sizes. **(Figure 1 attached)**

9. Table 3: Line 349) Table 3 shows that the visit time at provider increases for each subsequent visit, not the travel time - the travel time only increases between 2nd and 3rd visit

**Response:**
Thank you for this helpful observation. We appreciate the reviewer’s attention to detail, which has helped improve the accuracy of our reporting. We agree with the reviewer’s assessment. The text has been revised to accurately reflect the data presented in **Table 3**. Specifically, we now note that the **visit time** at each provider increases with each subsequent visit, while **travel time** remains relatively stable between the first and second visits and only increases between the second and third provider.

**Page 18; Line 355-358**

9. Discussion: 543/544 I would propose that “worsening condition with potentially worse treatment outcome (e.g. long term-disability) of the affected person and ongoing transmission” should be the first concerns before MDR TB (as long as no TB drugs are use pre-diagnostic, the development of drug resistance is unlikely) – DR TB becomes an issue when Pharmacies/ drug sellers or informal providers provide TB drugs of insufficient strengths and unproven combinations. I would suggest referencing the immediate risks to patients and programme first, as they are touched upon by the respondents already (“I got worse” “I lost all my possessions”) – DR-TB does not seem the main issue in your catchment area, as only 3 out of your 261 participants were diagnosed with/ treated for DR-TB (1%)

**Response:** Thank you for this valuable suggestion. We agree that emphasizing the **immediate risks** to patients and the health programme such as worsening of condition, long-term disability, ongoing transmission should precede discussion of MDR-TB in the manuscript. We also acknowledge that in our study area DR‑TB appears relatively rare (1% of participants), which means other harms may be more urgent or visible to the affected individuals.

**Page 29, Line 554-557; Line 595-597**

11. COVID-19 Line 599-602: by the very nature of things, it is hard to distinguish COVID-19 from TB just by symptomatic assessment – no education programme with change this. The key is that patients expressed fear of being diagnosed with COVID-19 and then having to face isolation and additional stigma. Therefore the 2 logical conclusions would be to a) ensure that anyone presenting with respiratory symptoms is tested for both TB and COVID-19; b) public health emergency measures are designed in a way that they do not frighten people suffering from other diseases away from accessing services. Policy makers, service providers and the public need the right information and stigma prevention measures.

**Response:** Thank you for this valuable insight. We agree that symptom overlap between COVID-19 and TB makes it inherently difficult to distinguish the two based on clinical presentation alone, and that no amount of patient education can fully resolve this diagnostic uncertainty. We also acknowledge your important point that fear of a COVID-19 diagnosis and the resulting stigma, isolation, or other consequences was a key barrier to care-seeking during the pandemic. We have revised the manuscript to reflect this nuance more accurately and to highlight the need for integrated testing, appropriate risk communication, and stigma-sensitive public health strategies, as you rightly suggest. **Page 32; Line 627-635**
